# A multiplex xTAG assay for the simultaneous detection of five chicken immunosuppressive viruses

**DOI:** 10.1186/s12917-018-1663-1

**Published:** 2018-11-15

**Authors:** Feng Cong, Yujun Zhu, Jing Wang, Yuexiao Lian, Xiangnan Liu, Li Xiao, Ren Huang, Yu Zhang, Meili Chen, Pengju Guo

**Affiliations:** 1 Guangdong Laboratory Animals Monitoring Institute and Guangdong Provincial Key Laboratory of Laboratory Animals, Guangzhou, 510633 China; 2 Guangdong Provincial Key Laboratory of Zoonosis Prevention and Control, College of Veterinary Medicine, South China Agricultural University, Guangzhou, 510640 China

**Keywords:** Simultaneous detection, Multiplex xTAG assay, Chicken anemia virus, Avian reovirus, Chicken immunosuppressive viruses

## Abstract

**Background:**

Chicken anemia virus (CAV), avian reovirus (ARV), infectious bursal disease virus (IBDV), Marek’s disease virus (MDV) and reticuloendotheliosis virus (REV) all cause immunosuppressive disease in birds through vertical or horizontal transmission. Mixed infections with these immunosuppressive pathogens lead to atypical clinical signs and obstruct accurate diagnoses and epidemiological investigations. Therefore, it is essential to develop a high-throughput assay for the simultaneous detection of these immunosuppressive viruses with high specificity and sensitivity. The aim of this study was to establish a novel method using a RT-PCR assay combined with fluorescence labeled polystyrene bead microarray (multiplex xTAG assay) to detect single or mixed viral infections.

**Results:**

The results showed that the established xTAG assay had no nonspecific reactions with avian influenza virus (AIV), infectious bronchitis virus (IBV), newcastle disease virus (NDV), infectious laryngotracheitis virus (ILTV), *Mycoplasma gallisepticum* (MG) and *Mycoplasma synoviae* (MS). The limit of detection was 1.0 × 10^3^ copies/μL for IBDV and 1.0 × 10^2^copies/μL for the other four viruses. Ninety field samples were tested and the results were confirmed using conventional RT-PCR methods. The detection results of these two methods were 100% consistent. The established multiplex xTAG assay allows a high throughput and simultaneous detection of five chicken immunosuppressive viruses.

**Conclusion:**

The multiplex xTAG assay has been showed to be an additional tool for molecular epidemiology studies of five chicken immunosuppressive viruses in the poultry industry.

**Electronic supplementary material:**

The online version of this article (10.1186/s12917-018-1663-1) contains supplementary material, which is available to authorized users.

## Background

Immunosuppression caused by viral infections is a common syndrome in the poultry industry. The result is temporary or permanent immune suppression, leaving the flock highly susceptible to other pathogenic agents. Young bird flocks are susceptible to five main immunosuppressive viruses, such as avian reovirus (ARV), chicken anemia virus (CAV), infectious bursal disease virus (IBDV), Marek’s disease virus (MDV) and reticuloendotheliosis virus (REV) [1–11]. Immunosuppression often leads to considerable losses in poultry production including a negative impact on vaccinations with low protection rates. Immunosuppression is especially problematic because primary disease control strategies frequently rely on efficient vaccines. In addition, several reports showed that co-infection with two or more immunosuppressive viruses either experimentally or through natural exposure enhanced the pathogenicity [5, 7, 8, 12–16]. Therefore, a rapid and cost-effective early diagnostic method would help prevent transmission within the farm.

Virus isolation and electron microscopy is the current standard for virus identification. However, these processes are time and labor intensive and require technical proficiency in these methods. Serological tests are sufficient to separate infected birds and are widely used on poultry production farms although immunosuppressed animals may test as falsely negative. Molecular biology techniques are now in use for characterization of viral pathogens. Conventional PCR and RT-PCR tests detect specific genes from target viral genomes. Agarose gel electrophoresis-based detection is insensitive compared with real-time and multiplex PCR/RT-PCR assays [17–19]. These latter approaches provide a fast and effective alternative with good sensitivity and specificity but the number of fluorophores is limited for multiplex real-time PCR instruments.

An alternative to these procedures is the commercial MagPlex-TAG system that enables high-throughput detection of multiple analytes in a single reaction. This allows simultaneous detection of viral target in a mixed infection [20, 21]. The method involves specific primers designed for target sequence amplification by PCR. The amplicons are detected by incubations with beads and streptavidin-R-phycoerythrin. In the assay reader, lasers identify the bead’s color set and the phycoerythrin reporter dye. In this study, a multiplex xTAG assay was established to distinguish five avian pathogens.

## Results

### Specificity, sensitivity and reproducibility tests

**Fig. 1:**
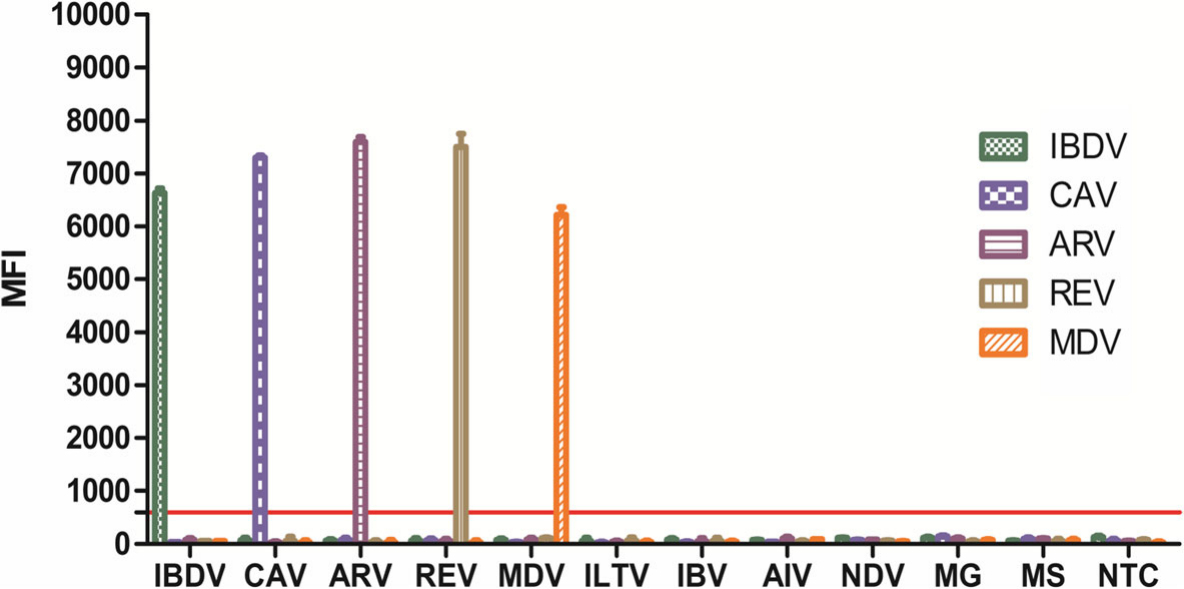
Result of the specificity testing. Viral target specificity was tested in a multiplex mode using target-specific primers. The targets of IBDV, CAV, ARV, REV and MDV were tested against each other and the non-target viral pathogens: avian influenza virus (AIV), infectious bronchitis virus (IBV), newcastle disease virus (NDV), infectious laryngotracheitis virus (ILTV), Mycoplasma gallisepticum (MG) and Mycoplasma synoviae (MS). The cut-off value was 600, defined as the mean of the net MFI from negative PCR controls with three times of standard deviation (MFI +3SD). All the samples were performed in triplicate

The specificity of each pair of target primers for viral sequence amplification was examined using DNA and RNA templates by both the multiplex xTAG assay and conventional PCR. There were no cross-reactions with avian influenza virus (AIV), infectious bronchitis virus (IBV), newcastle disease virus (NDV), infectious laryngotracheitis virus (ILTV), *Mycoplasma gallisepticum* (MG) and *Mycoplasma synoviae* (MS) (see Fig. 1 and Additional file 1: Table S1).

**Fig. 2:**
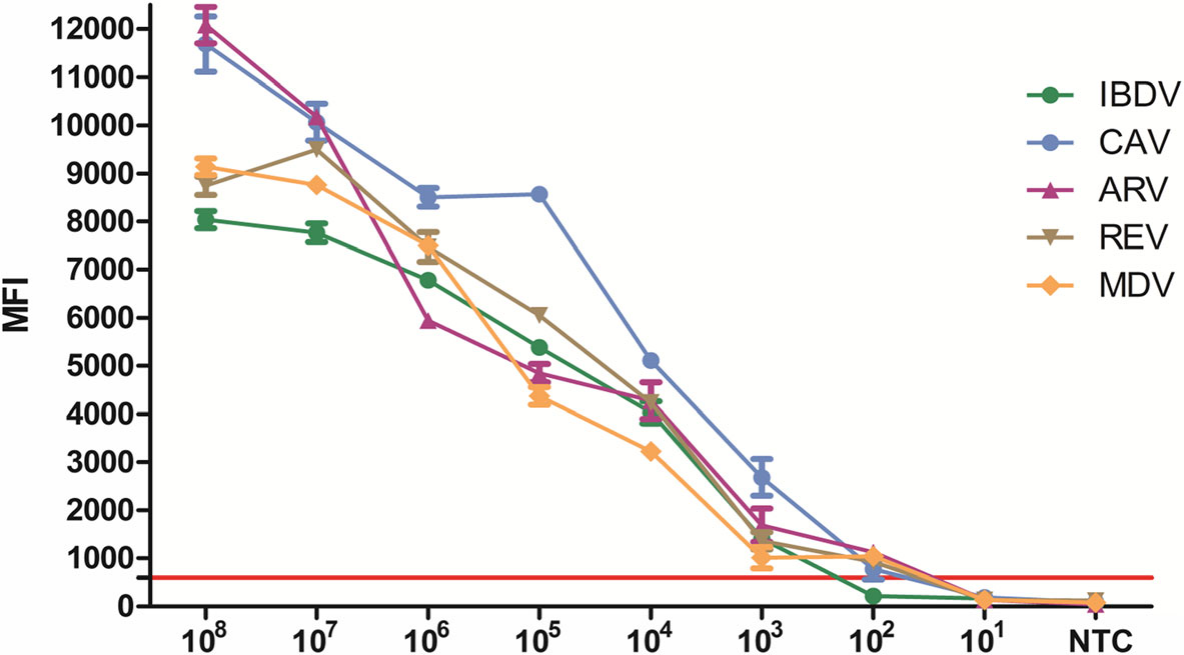
Result of Sensitivity testing. The concentration of DNA or RNA standards ranged from 1 × 10^8^ copies/ul to 1 × 10^1^ copies/ul. The cut-off value was 600. the limit of detection was 1 × 10^3^ copies/ul for IBDV and 1 × 10^2^ copies/ul for CAV, ARV, REV, MDV. All the samples were performed in triplicate

The minimum requirement for detection of target sequences was determined as the median fluorescence intensity (MFI) of the replicates above the cut-off value from the highest plasmid or RNA dilution. The limit of detection was 1.0 × 10^3^copies/μL RNA for IBDV and 1 × 10^2^copies/μL for ARV, CAV, MDV and REV. The sensitivity of multiplex xTAG assay for CAV was ten-fold higher than conventional PCR and had similar sensitivity for the other four viruses (see Fig. 2 and Table 1).

**Table 1:** The average of the MFI values with SD of sensitivity test by the xTAG assay and conventional PCR/RT-PCR

Sample (copies/ul)	IBDV		CAV		ARV		REV		MDV	
	xTAG assay		xTAG assay		xTAG assay		xTAG assay		xTAG assay	
	MFI values ±SD	RT-PCRP/N	MFI values ±SD	RT-PCRP/N	MFI values ±SD	RT-PCRP/N	MFI values ±SD	RT-PCRP/N	MFI values ±SD	RT-PCRP/N
10^8^	8035 ± 178	+	11,687.5 ± 574	+	12,380 ± 382	+	8742.5 ± 191	+	9143.5 ± 172	+
10^7^	7764 ± 84	+	10,063.5 ± 279	+	10,208 ± 465	+	9302 ± 170	+	8702 ± 152	+
10^6^	6782 ± 138	+	8503 ± 191	+	5944 ± 119	+	7470.5 ± 310	+	7496 ± 169	+
10^5^	5380.5 ± 110	+	8567 ± 185	+	4803 ± 135	+	5938 ± 97	+	4350 ± 198	+
10^4^	4034 ± 230	+	5107.5 ± 72	+	4277 ± 382	+	4233 ± 134	+	3227 ± 157	+
10^3^	1402 ± 38	+	2680.5 ± 122	+	1683 ± 347	+	1358.5 ± 182	+	1806.5 ± 220	+
10^2^	215 ± 184	–	767.5 ± 206	+	1121 ± 26	+	924 ± 37	+	1039 ± 128	+
10^1^	167 ± 138	–	191.5 ± 139	–	137 ± 38	–	141.5 ± 46	–	138.5 ± 59	–
blank	78 ± 45	–	89 ± 17	–	48 ± 35	–	128 ± 83	–	85 ± 21	–

*MFI* median fluorescent intensity

+: positive (P)

-: negative (N)

DNA or RNA standards at the range of 1 × 10^5^ and 1 × 10^7^ copies/μL were prepared for reproducibility test. The inter-assay variation fell between 1.4 to 4.6%. The intra-assay variations were blind tested in duplicates by another user using three parallel reactions at 1 × 10^5^ and 1 × 10^7^ copies/μL. These variations ranged from 1.2 to 3.3% (Table 2).

**Table 2:** CV% of reproducibility tests

	Copies/ uL	Intra-assay MFI			CV(%)	Inter-assay MFI			CV(%)
		1	2	3		1	2	3	
CAV	1 × 10^5^	8684	8353	8664	2.1	8567	8216	8317	2.2
	1 × 10^7^	10,177.5	9745	10,268	2.7	10,063.5	9602	10,057	2.6
MDV	1 × 10^5^	4374.5	4536	4142	4.6	4350	4637	4575	3.3
	1 × 10^7^	8761	8816	8529	1.8	8702	8923	8627	1.8
IBDV	1 × 10^5^	5420.5	5465.5	5255.5	2.1	5380.5	5268	5528	2.4
	1 × 10^7^	7764	7848	7680	1.4	7764	7901	7658	1.6
ARV	1 × 10^5^	4947	4678	4784	2.8	4803	4917	4639	2.9
	1 × 10^7^	10,082	10,723.5	9819	4.6	10,208	9957	10,084	1.2
REV	1 × 10^5^	6051	5874	5890	1.6	5938	5987	5703	2.5
	1 × 10^7^	9499	9213.5	9196	1.8	9302	9248.5	9645	2.3

### Application of field samples

Among 90 field samples tested, six samples were identified as ARV positive and one specimen as IBDV positive. Eight samples were MDV positive only, fourteen samples were CAV/MDV co-infections, six were REV positive only, one was CAV/REV co-infections and one case was a CAV/MDV/REV mixed infection. The rest of samples were CAV positive with an occurrence rate of 67.8%. DNA sequence analysis verified that the amplified target fragments were identical to specific viral sequences (Table 3 and Additional file 2: Table S2).

**Table 3:** Screening results for 90 clinical samples using the xTAG assay

Sample	CAV	MDV	IBDV	ARV	REV	Sample	CAV	MDV	IBDV	ARV	REV
T1	+++	–	–	–	–	T46	++	++	–	–	–
T2	+++	–	–	–	–	T47	++	+	–	–	–
T3	+++	–	–	–	–	T48	++	++	–	–	–
T4	+++	–	–	–	–	T49	++	++	–	–	–
T5	+++	–	–	–	–	T50	++	+	–	–	–
T6	+++	+	–	–	–	T51	++	–	–	–	–
T7	+++	–	–	–	–	T52	++	+	–	–	–
T8	+++	–	–	–	–	T53	++	++	–	–	–
T9	+++	–	–	–	–	T54	++	++	–	–	–
T10	+++	–	–	–	–	T55	++	++	–	–	–
T11	+++	–	–	–	–	T56	–	–	–	–	–
T12	+++	–	–	–	–	T57	–	–	–	–	–
T13	–	++	–	–	–	T58	–	–	–	–	–
T14	+++	+	–	–	–	T59	–	–	–	–	–
T15	+++	–	–	–	–	T60	–	–	–	–	–
T16	+	++	–	–	–	T61	–	–	–	–	–
T17	+++	–	–	–	–	T62	–	–	–	++	–
T18	–	+	–	–	–	T63	–	–	–	–	++
T19	+++	–	–	–	–	T64	–	–	–	–	++
T20	++	–	–	–	–	T65	–	–	–	–	++
T21	+++	–	–	–	–	T66	–	–	–	–	++
T22	+++	–	–	–	–	T67	–	–	–	–	++
T23	+++	–	–	–	–	T68	–	–	–	–	++
T24	+	–	–	–	–	T69	–	–	–	–	–
T25	+++	–	–	–	–	T70	–	–	–	–	–
T26	+++	–	–	–	–	CS1	+++	–	–	–	–
T27	+++	–	–	–	–	CS2	+++	+	–	–	–
T28	–	+++	–	–	–	CS3	+++	–	–	–	–
T29	+++	–	–	–	–	CS4	–	+	–	–	–
T30	+++	–	–	–	–	CS5	++	–	–	–	–
T31	++	–	–	–	–	CS6	++	–	–	–	–
T32	+++	–	–	–	–	CS7	–	++	–	–	–
T33	++	–	–	–	–	CS8	+++	++	–	–	–
T34	++	–	–	–	+	CS9	++	–	–	–	–
T35	+++	–	–	–	–	CS10	+++	–	–	–	–
T36	+++	–	–	–	–	CS11	+++	–	–	–	–
T37	+++	++	–	–	+	CS12	–	–	–	+	–
T38	+++	–	–	–	–	CS13	–	–	–	++	–
T39	+++	–	–	–	–	CS14	–	++	–	–	–
T40	–	–	–	++	–	CS15	–	–	++	–	–
T41	–	–	–	+	–	CS16	+	–	–	–	–
T42	–	–	–	++	–	CS17	++	–	–	–	–
T43	–	+	–	–	–	CS18	++	–	–	–	–
T44	+++	–	–	–	–	CS19	++	–	–	–	–
T45	–	++	–	–	–	CS20	++	–	–	–	–

*T* tissue specimens, *CS* cloacal swabs;

+++: strong positive (MFI > 5 × cutoff);

++: positive (3 × cutoff <MFI < 5 × cutoff);

+: weak positive cutoff (MFI < 3 × cutoff);

-: negative (MFI < cutoff)

### Artificial mixed infection analysis

**Fig. 3:**
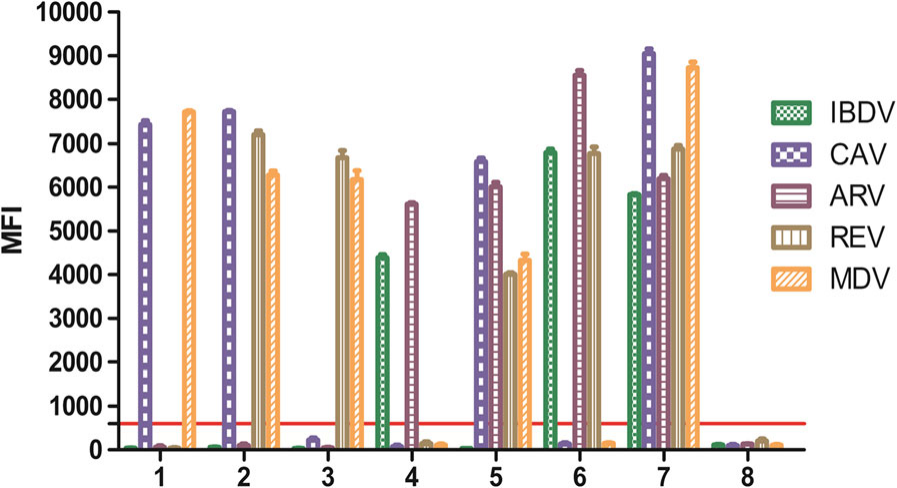
Result of artificial mixed infection testing. Each bar represents the average median fluorescence intensity (MFI) of triplicate samples with standard deviation. The cut-off value was 600. 1: CAV and MDV positive sample; 2: CAV, MDV and REV positive sample; 3: MDV and REV positive sample; 4: IBDV and ARV positive sample; 5: CAV, MDV, ARV and REV positive sample; 6: IBDV, ARV and REV positive sample; 7: IBDV, ARV, REV, CAV, MDV positive sample; 8: negative control

To evaluate potential interference of the xTAG assay in multiplex infection, artificial mixed samples were obtained by spiking. Three samples were prepared as double infections and two samples were made as triple infection. Meanwhile one sample was prepared as CAV/MDV/ARV/REV mixed infection and one was CAV/MDV/ARV/REV/IBDV co-infection (Fig. 3). The results showed that the developed multiplex xTAG assay is no potential interference when multiple viral targets were present.

## Discussion

Immunosuppression among chicken flocks causes significant economic losses in the poultry industry. These immunosuppressed birds are highly susceptibility to secondary infections with other viruses or bacteria. Mixed infections with two or more pathogens would obscured typical clinical signs and increase the difficulty for accurate diagnosis [1, 22]. Mixed viral infections are common in bird flocks and infections with CAV, IBDV, REV, ARV and MDV all lead to a decline of humoral and cell-mediated immunity [1, 3, 4, 7, 9]. An assay that can detect multiple viruses simultaneously would greatly enhance clinical diagnoses. In comparison with conventional PCR and ELISA, the multiplex PCR assay allows multiplexing and reduces reagent consumption and material preparation time. However, multiple primer pair interactions in the multiplex PCR assay can adversely affect accuracy [23].

The multiplex xTAG assay established in this study is available to support high-throughput and simultaneous molecular diagnosis of five immunosuppressive viral pathogens in a cost-effective manner with high specificity and sensitivity. Target selection is important to provide a successful diagnostic method [20]. For example, although CAV sero-positive distribution in chickens is worldwide, there is no significant genetic difference for isolates from various regions and only one serotype has been classified [24]. Based on sequence alignments, the *VP1* gene was selected for CAV detection due to its function as main structural protein for capsid assembly. On the other hand, the IBDV strains have multiple presentations of antigenicity and virulence. A very virulent IBDV emerged three decades ago and had a high mortality rate. Therefore, the *VP2* gene was the better choice for the multiplex xTAG assay because it is able to induce neutralizing antibody and possesses a highly conserved region that can differentiate between vaccine and wild type strains [25]. For MDV, the *MEQ* gene is a major latency antigen expressed in the oncogenic serotype 1 but not serotypes 2 and 3 [9, 26]. MEQ is ideal to distinguish between these serotypes in the PCR assay. The *ENV* gene in REV encodes a glycoprotein that comprises the binding pocket for neutralizing antibody. The *σC* protein expressed by the *S1* gene in ARV mediates viral attachment and antibody-specific neutralization [5, 27–29]. Thus the conserved regions in these two genes were chosen for primer design.

The PH value of the hybridization buffer is also important to MFI value. When the PH value is 8.0, the MFI value of the negative sample is the lowest, the MFI value of the positive sample is the highest. When the PH value is not optimal, the cut-off value is higher, which lead to the false negative results.

The specificity and sensitivity of xTAG-multiplex PCR assay were determined in this study and it was found no cross-reactions with other common chicken viral pathogens, indicating that xTAG-multiplex PCR assay had a high specificity. The limit of detection of xTAG-Multiplex PCR assay for IBDV was 1.0 × 10^3^copies/μL while the limit of detection for the other four viruses was > 1.0 × 10^2^copies/μL. Compared to conventional PCR, the sensitivity of xTAG multiplex PCR for CAV was ten-fold higher while it remains similar sensitivity for the other four viruses.

Among 90 field samples, the detection results using Multiplex xTAG assay were 100% consistent with those tested by conventional PCR assay, demonstrated that the multiplex xTAG assay is high-throughput and suitable for molecular epidemiology studies of five immunosuppression viruses. More than 60% CAV positive rate revealed that the viral infection control strategies on the farm were not fully successful. Five cases of mixed infections of CAV and MDV were found based on the result of xTAG assay, which may induce a synergistic effect on infection and cause severe immunosuppression. A high positive infection rate of CAV also suggests vaccination failure. An annual veterinary evaluation would do much to draw attention to potential problems of immunosuppression and vaccine failure.

## Conclusion

The multiplex xTAG assay can perform the simultaneous detection of five avian immunosuppressive viruses in one reaction. This will enhance clinical diagnoses and epidemiological investigations.

## Methods

### Viruses and field samples

The virus strains IBDV B87, REV C15 and the vaccine strain MDV 814 were purchased from Guangdong Animal Epidemic Prevention and Material Reserve Center. Cell culture adapted strains CAV GD-2014, ARV GD-2, Avian influenza virus (AIV) H7N2 subtype, newcastle disease virus (NDV) F48E9 strain, infectious bronchitis virus (IBV) Massachusetts 41, infectious laryngotracheitis virus (ILTV) N-71851 strain (ATCC VR-783) were maintained in our laboratories. *Mycoplasma gallisepticum* (MG) and *Mycoplasma synoviae* (MS) were purchased from the China Veterinary Culture Collection.

90 chicken samples (cloacal swabs, livers, spleens and kidneys) from a poultry farm in Guangdong province were submitted to our laboratory at Guangdong Laboratory Animals Monitoring Institute for routine animal health surveillance.

### Nucleic acid extraction

Frozen chicken organ samples were homogenized for 15–30s before use. Cultured cell harvests were frozen and thawed several times followed by low speed centrifugation. The supernatants were stored at − 80 °C until use. Lyophilized vaccine samples were suspended in phosphate buffered saline following the manufacturer’s protocol. Viral genomic DNA and RNA were extracted from homogenates and lysed cell culture supernatants using TIANamp Virus DNA/RNA Kit following the company’s protocol (DP202, Tiangen Biotech, BeiJing). The samples after extraction were eluted in 70 μL RNase-free ddH_2_O and stored at − 80 °C.

### Primer design and selection

Full-length viral genome sequences were retrieved from the National Center for Biotechnology Information (NCBI) GenBank database (https://www.ncbi.nlm.nih.gov/) and each primer pair was designed based on multiple sequence alignment from Clustal W implemented in Geneious version 5.4.3 (Biomatters, Auckland, NZ). Primer oligomers were evaluated using Primer Premier 5.0 software (www.premierbiosoft.com).

xTAG assay uses target-specific primers which have linker overhangs (TAG sequences) on one primer which allow the PCR products to attach to a differently colored bead sets (xTAG beads, Luminex, Austin, TX, USA) and biotin on the other primer so that the PCR products contain biotin. Attached to each differently colored bead is an anti-TAG sequence which only binds to the complementary TAG sequence on the primer. After PCR amplification, color-coded beads were hybridized with the target PCR products via the TAG and anti-TAG complementarity. And then streptavidin-R-phycoertyhrin was added to bind to the biotin on the PCR amplicons. The intensity of fluorescence of the beads and R-phycoerythrin was determined by Luminex® 200 instrument. All primer sets were synthesized and purified by polyacrylamide gel electrophoresis (PAGE) (Invitrogen, Guangzhou, China). The gene targets were CAV (*VP1* gene), MDV (*MEQ* gene), REV (*ENV* gene), ARV (*S1* gene) and IBDV (*VP2* gene) (Table 4).

**Table 4:** Primer pairs used in this study

Targeted virus	Forward primers (5′-3′)	Reverse primers (5′-3′)	Amplicon size (bp)	Bead region	Source
CAV	ATACTTTACAAACAAATAACACAC-spacer18-GCTCTCCAAGAAGATACTCCA	GCATTCGCGCAGCCACAC	149	19	this study
MDV	TACTTAAACATACAAACTTACTCA-spacer18-CCCATTCCCTCTTCTGCC	GCTGAGCGTAAACCGTC	114	65	this study
IBDV	TACTTCTTTACTACAATTTACAAC-spacer18-ATGCGGAGCCTTCTGA	ATTAGCCCTGACCCTGTG	128	15	this study
ARV	ACTTATTTCTTCACTACTATATCA-spacer18-GGATTCCGTCTCCATTCT	GAGTTTCCGTCAACCGTA	102	34	this study
REV	CTTAAACTCTACTTACTTCTAATT-spacer18-GACTGCCTTGTGACTGCT	ACTCCCACTGTTGTCTAAATC	153	56	this study
CAV	GACTGTAAGATGGCAAGACGAGCTC	GGCTGAAGGATCCCTCATTC	675	/	Caterina et al., 2004 [17]
IBDV	AGCCTTCTGATGCCAACAAC	ATCTGTCAGTTCACTCAGGC	365	/	Caterina et al., 2004 [17]
ARV	GGTGCGACTGCTGTATTTGGTAAC	AATGGAACGATAGCGTGTGGG	532	/	Caterina et al., 2004 [17]
MDV	GTGATGGGAAGGCGATAGAA	TCCGCATATGTTCCTCCTTC	225	/	Cao et al., 2013 [30]
REV	AATGGTTGTAAAGGGCAGAT	CTCCTCTCACTGCCAATCT	200	/	Cao et al., 2013 [30]

### Multiplex nucleic acid amplification and hybridization assay

The nucleic acid amplifications were performed using the OneStep RT-PCR Kit (Qiagen, Valencia, CA, USA; Cat. no. 210212). Each 20 μL reaction contained 1 μL nucleic acid template, 0.8 μL OneStep RT-PCR Enzyme Mix, 250 nM of each primer, 0.8 μL dNTP mixes and 4 μL OneStep RT-PCR buffer. Cycling conditions for the multiplex PCR were 50 °C for 30 min, 95 °C for 15 min, 40 cycles of 94 °C for 25 s, 55 °C for 25 s, and 72 °C for 20 s and 72 °C for 10 min. After reaction completion, 5 μl of the reaction mix was added to 75 μl of streptavidin-R-phycoerythrin (SAPE, Life Technologies GmbH) and 20 μl beads (approximately 2500 beads). The hybridization process was carried out at 37 °C for 30 min in a thermal cycler. After the hybridization reaction, the products were analyzed on the Luminex® 200 instrument.

### Data analysis and cut-off value determination

The median fluorescent intensity (MFI) was calculated based on conditions suggested by the manufacturer and at least 50 beads were counted for each bead set in a single experiment. The data was analyzed using the Luminex xPONENT software 3.1. Negative controls containing all the hybridization components except target nucleic acid were used in each experiment to reduce background interference. The cut-off value was defined as the mean of the net MFI from negative PCR controls plus three times of standard deviation (MFI + 3SD).

### Assay sensitivity and specificity tests

Sensitivity testing was determined by using 10-fold serial dilutions of DNA or RNA standards as assay templates. PCR products of the five viral PCR products were cloned in plasmid vector pGEM T Easy (Promega, Madison, WI. USA). The constructs of IBDV, REV and ARV were in vitro transcribed using a RiboMax Large Scale RNA production system T7 (Promega) according to the manufacturer’s instructions. RNase-free DNase (40 U) (Promega) was used for processing. Trizol LS reagent (Invitrogen, Carlsbad, CA) was used for RNA isolation to eliminate DNA interference. RNA concentrations were estimated by UV spectrophotometry from the average of five repeats. The copy numbers of DNA or RNA standards were calculated based on the nucleic acid concentration and molecular weight. Assay sensitivity was compared to conventional PCR using 10-fold serial dilutions of DNA or RNA standards. All the samples were performed in triplicate.

Assay specificity was tested using samples of non-target viruses in the reactions, such as AIV, IBV, NDV, ILTV, MG and MS. PCR products from specificity experiments were excised from 1.0% agarose gels using an AxyPrep DNA gel extraction kit (Axygen Scientific, Inc., CA) and sequenced (Invitrogen, Guangzhou, China).

### Sample tests

Homogenates of organ and swab samples were used for RNA extraction and simultaneously tested for the presence of CAV, MDV, IBDV, REV and ARV using the multiplex xTAG assay and conventional PCR (Table 4) [30]. PCR products were sequenced. To further evaluate the xTAG assay, artificial mixed samples with combinations of the different viruses were tested.

## Additional files

Additional file 1: **Table S1.** The average of the MFI values with SD of specificity test by the xTAG and conventional PCR/RT-PCR assay. (DOCX 15 kb)

Additional file 2: **Table S2.** Results for clinical samples examined by the xTAG multiplex RT-PCR and conventional PCR/RT-PCR assay. (DOC 17 kb)

## Abbreviations

AIV: Avian influenza virus
ARV: Avian reovirus
CAV: Chicken anemia virus
IBDV: Infectious bursal disease virus
IBV: Infectious bronchitis virus
ILTV: Infectious laryngotracheitis virus
MDV: Marek’s disease virus
MG: Mycoplasma gallisepticum and
MS: Mycoplasma synoviae
NDV: Newcastle disease virus
REV: Reticuloendotheliosis virus
