# Supplementary material for: A multiplex xTAG assay for the simultaneous detection of five chicken immunosuppressive viruses
Source: BMC Vet Res. 2018 Nov 15;14:347. doi: 10.1186/s12917-018-1663-1 (PMC6238339; doi:10.1186/s12917-018-1663-1)
Supplement: Supplementary file 1 — Table S1. The average of the MFI values with SD of specificity test by the xTAG and conventional PCR/RT-PCR assay. (DOCX 15 kb) [file 12917_2018_1663_MOESM1_ESM.docx]

**Table S1** The average of the MFI values with SD of specificity test by the xTAG and conventional PCR/RT-PCR assay

| Virus | IBDV | | CAV | | ARV | | REV | | MDV | |
| --- | --- | --- | --- | --- | --- | --- | --- | --- | --- | --- |
|  | xTAG assay conventional  MFI values RT-PCR  ±SD P/N | | xTAG assay conventional  MFI values RT-PCR  ±SD P/N | | xTAG assay conventional  MFI values RT-PCR  ±SD P/N | | xTAG assay conventional  MFI values RT-PCR  ±SD P/N | | xTAG assay conventional  MFI values RT-PCR  ±SD P/N | |
| IBDV  CAV  ARV  REV  MDV  ILTV  IBV  AIV  NDV  MG  MS  NTC | 6691±125  33±16  81±14  24±11  61±27  111±44  104±28  68±13  110±38  132±52  68±26  105±41 | +  -  -  -  -  -  -  -  -  -  -  - | 39±19  7329.5±138  52.5±28  43.5±15  33±10  38.5±9  33±16  43±20  69±31  126±46  75±21  38±17 | -  +  -  -  -  -  -  -  -  -  -  - | 98.0±51  46±22  7662.5±199  86±15  56±18  56±13  91±35  77±30  82±23  88±47  110±38  55.5±23 | -  -  +  -  -  -  -  -  -  -  -  - | 59±31  62±26  56±17  7326±248  107±42  123±36  98±40  43±11  60±27  35±8  86±22  67.5±14 | -  -  -  +  -  -  -  -  -  -  -  - | 60±19  59±23  37±10  117±44  6320±152  132±32  104±26  66.5±15  29.5±21  90±51  45±21  68±43 | -  -  -  -  +  -  -  -  -  -  -  - |

MFI: median fluorescent intensity

+: positive (P)

-: negative (N)
