# Supplementary material for: A multiplex xTAG assay for the simultaneous detection of five chicken immunosuppressive viruses
Source: BMC Vet Res. 2018 Nov 15;14:347. doi: 10.1186/s12917-018-1663-1 (PMC6238339; doi:10.1186/s12917-018-1663-1)
Supplement: Supplementary file 2 — Table S2. Results for clinical samples examined by the xTAG multiplex RT-PCR and conventional PCR/RT-PCR assay. (DOC 17 kb) [file 12917_2018_1663_MOESM2_ESM.doc]

**Table S2** Results for clinical samples examined by the xTAG multiplex RT-PCR and conventional PCR/RT-PCR assay

| Virus | No. of samples  xTAG-multiplex PCR | No. of samples  RT-PCR/PCR: |
| --- | --- | --- |
|  | Positive Negative | Positive Negative |
| CAV | 69 21 | 69 21 |
| MDV | 23 67 | 23 67 |
| ARV | 6 84 | 6 84 |
| REV | 8 82 | 8 82 |
| IBDV | 1 89 | 1 89 |
